# Bilateral Synovial Cysts as a Rare Cause of Myelopathy in a 38-year-old Woman

**DOI:** 10.7759/cureus.5377

**Published:** 2019-08-13

**Authors:** Martina L Mustroph, Christian D Cerecedo-Lopez, Michael Groff, Hasan A Zaidi

**Affiliations:** 1 Neurosurgery, Brigham and Women's Hospital, Boston, USA; 2 Neurosurgery, Harvard Medical School, Boston, USA

**Keywords:** bilateral, case report, synovial cyst, spine, cervical, myelopathy, neurosurgery

## Abstract

Synovial cysts are rare, and they occur even more rarely bilaterally or in the cervical spine. A 38-year-old previously healthy female presented with acute onset of numbness and tingling down her arms and weakness in her legs, which progressed steadily over 2-3 weeks to include significant gait disturbance. She denied bowel or bladder symptoms, saddle anesthesia, night sweats, weight loss, fever, or chills. MRI spine revealed a C7-T1 extradural mass consistent with bilateral synovial cysts emanating from bilateral neuroforamina resulting in critical spinal cord compression with T2 signal change in the cord. There was questionable patch enhancement after gadolinium contrast. The patient underwent C7-T1 laminectomies and partial bilateral medial facetectomies with excision of the cysts. Intraoperative cultures unexpectedly grew Staphylococcus aureus, suggesting superinfection of cysts. The patient recovered neurologic function postoperatively and was discharged on a 6-week course of IV antibiotics. We report and discuss the clinical presentation, pathogenesis, and neuroradiological findings in an adult case of bilateral synovial cysts at the C-T-spine junction. Immediate resection at symptom onset is indicated due to the good clinical outcome following resection and the real risk of paralysis if cysts are not excised in a timely fashion.

## Introduction

Spinal synovial cysts (SSCs) are pathologic collections of synovial fluid and/or other materials lined by synovial membrane located close to the facet joints [1]. SSCs occur more commonly in the lumbar and thoracic spine, with only a few reports of cervical spinal synovial cysts (CSSCs) [2]. We present a case of bilateral CSSCs in a 38-year-old female.

## Case presentation

**Figure 1:**
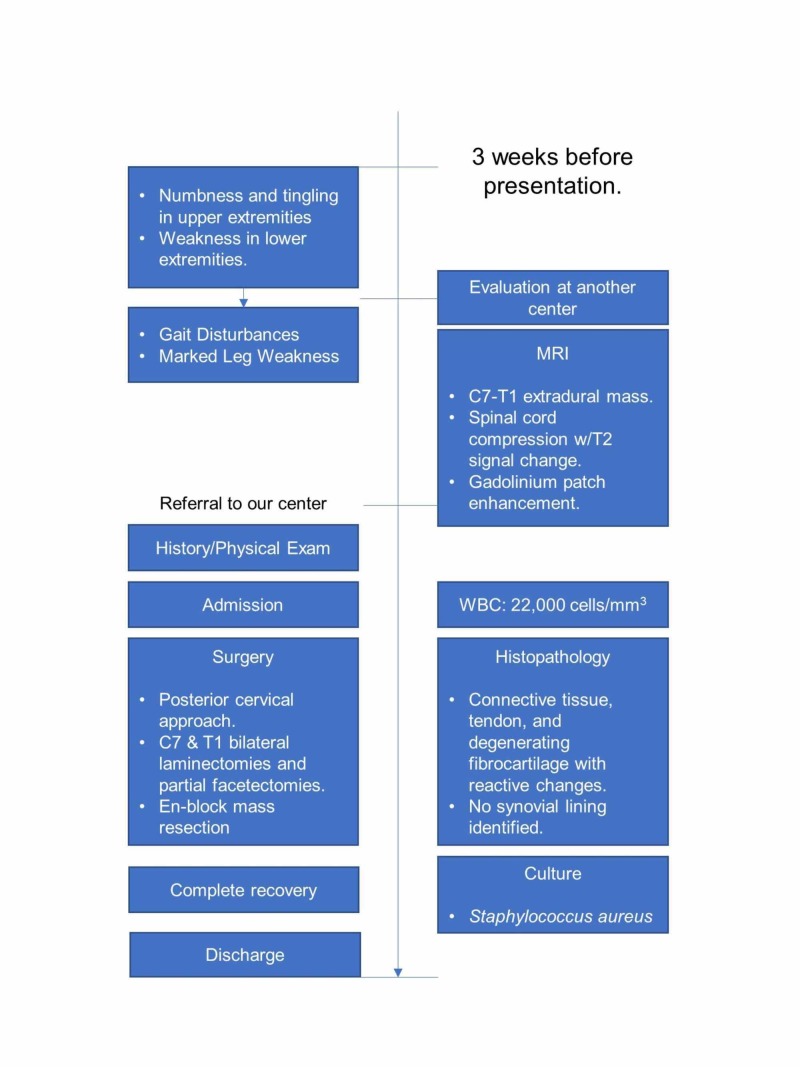
Timeline of Patient Care

**Figure 2:**
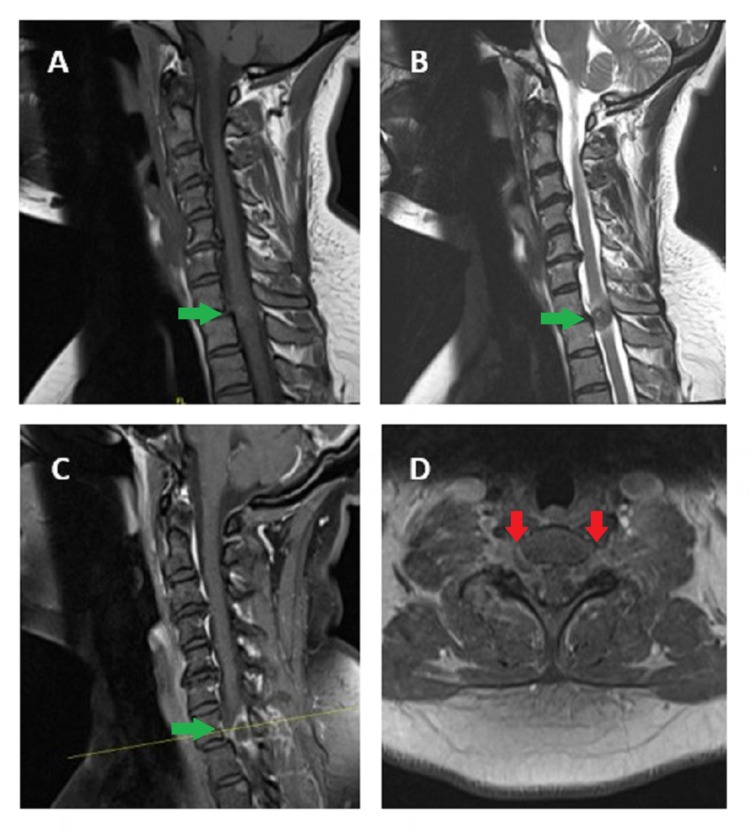
Plain and Enhanced Magnetic Resonance Imaging (MRI) (A) Sagittal T1-weighted MRI showing an extradural mass isointense to spinal cord at C7-T1 (green arrow), (B) Sagittal T2- weighted MRI showing an isointense mass (green arrow), (C) Sagittal postcontrast T1-weighted MRI showing a partially enhancing lesion with cord compression at C7-T1 (green arrow), (D) Axial postcontrast T1-weighted MRI showing bilateral foraminal nerve root compression at C7-T1 (red arrows)

A 38-year-old previously healthy Caucasian female presented with 2-3 weeks of numbness and tingling in her upper extremities and weakness in her lower extremities that progressed steadily to gait disturbances and marked leg weakness (Figure 1). MRI of the cervical spine revealed a C7-T1 extradural mass emanating from bilateral neuroforamina, resulting in critical spinal cord compression with T2 signal change in the cord at C7-T1. The mass exhibited questionable patch enhancement after gadolinium contrast (Figure 2). The patient presented to us for a second opinion.

Initial physical examination revealed a mild decrease in handgrip strength bilaterally (4/5). Deep tendon reflexes (DTRs) of the upper extremities were normal bilaterally (2+/4). Both lower extremities (LE) were 4/5 in strength with unremarkable DTRs (2+/4). Hoffman’s and Babinski reflexes were positive bilaterally. Sensory examination revealed normal light touch, vibration, proprioception, and pinprick sensation in all extremities.

A preliminary diagnosis of abscess was considered; however, the patient’s history-namely, the absence of predisposing risk factors such as IV drug use, immunocompromise, prior infections, and the absence of symptoms like fevers or chills-did not fit with a diagnosis of abscess. Laboratory analysis performed on the day of hospital admission revealed an elevated white blood cell count (22,000 cells/mm^3^).

The patient was admitted to our hospital, and an expedited decompression procedure with resection of the lesions and possible instrumented fusion was planned for hospital day-one. The risks, benefits, and side effects of surgery, disease progression, prognosis, and possible alternative treatment options were discussed with the patient.

**Figure 3:**
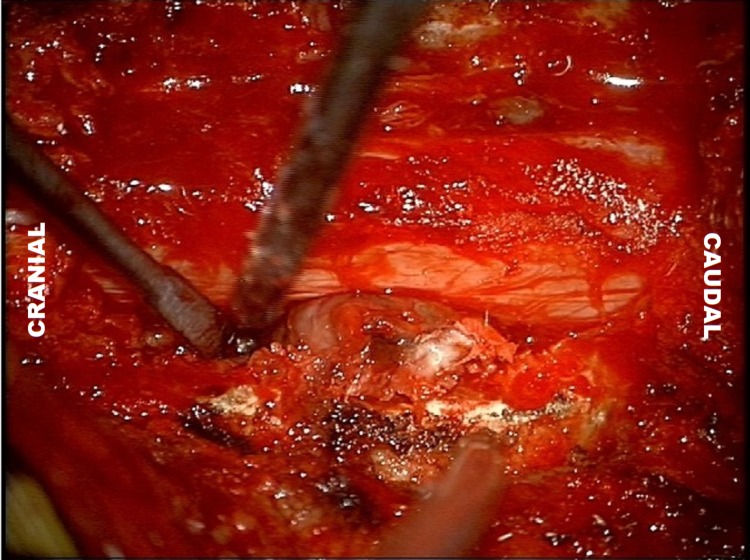
Intraoperative Photograph Intraoperative photograph showing an extradural extramedullary lesion emanating from facet joints bilaterally at C7-T1

Bilateral laminectomies and partial medial facetectomies were performed at C7 and T1 under general anesthesia in the prone position. Bilateral lesions that appeared to be emanating from the C7-T1 facet joints were identified (Figure 3), and careful dissection of a plane between the cervical spinal cord and the lesions was performed. On gross intraoperative examination, the lesions appeared to be synovial cysts with no evidence of purulent drainage. After the capsule was dissected, complete en bloc microscopic excision of the bilateral lesions was performed, and the lesions were removed as two separate pieces. Pieces (not whole cysts) were sent for histopathological examination, Gram stain, and cultures. Intraoperative blood loss was minimal. The patient received perioperative vancomycin 1500mg IV and ceftriaxone 2g IV. We decided against spinal instrumentation given the risk for persistent infection in the setting of new hardware placement, as well as the fact that only minimal facetectomies were necessary to access the lesion safely.

**Figure 4:**
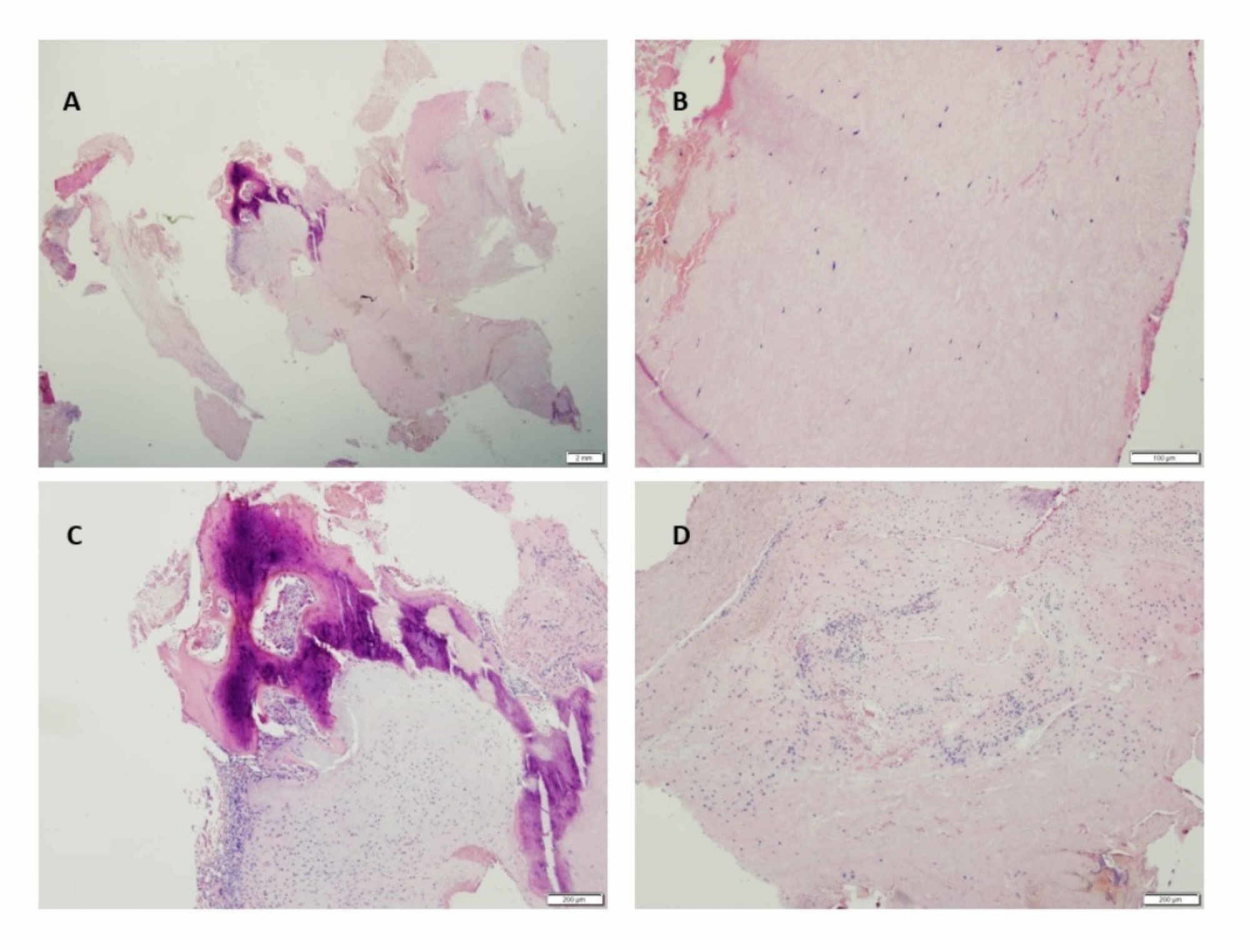
Pathology Specimen of C7-T1 Resected Lesion (A) Hematoxylin and eosin stain showing all connective tissue elements including bone, cartilage, and tendon; (B) Hematoxylin and eosin stain showing tendon; (C) Hematoxylin and eosin stain showing woven bone with reactive changes and fibrosis; (D) Hematoxylin and eosin stain showing degenerative fibrocartilage with reactive changes

Histopathological examination of the epidural C7-T1 masses revealed connective tissue, tendon, and degenerating fibrocartilage with reactive changes (Figure 4). Synovial cyst or lining was not identified but could not be excluded.

On postoperative day one, the patient received a four-day dexamethasone taper and Miami J collar to be worn until follow-up. Aerobic intraoperative wound cultures returned positive for Staphylococcus aureus susceptible to vancomycin. C-reactive protein was elevated (32.9mg/L), while procalcitonin was and remained normal (<0.08ng/mL). The patient was started on vancomycin IV 1.5g and cefazolin IV 2gm every eight hours. Blood cultures remained negative. The patient remained afebrile during her hospital stay.

**Figure 5:**
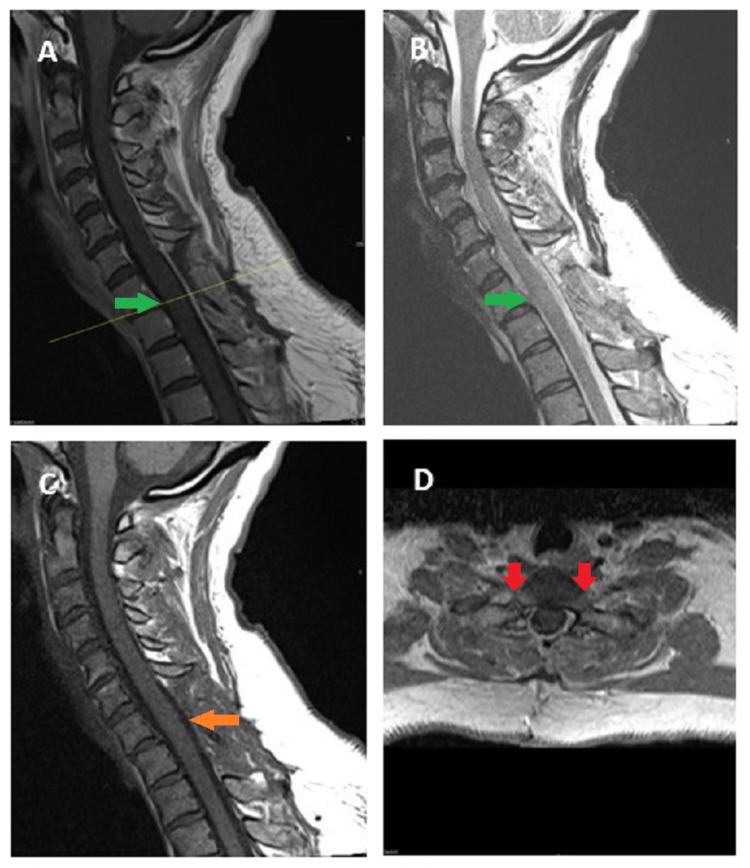
Post-Operative MRI after C7-T1 Laminectomies and Partial bilateral C7-T1 Facetectomies with Resection of Lesion (A) Sagittal T1-weighted MRI showing that the previously seen narrowed dural sac at C7-T1 related to the bilateral extradural foraminal lesions was decompressed (green arrow); (B) Sagittal T2-weighted MRI again shows interval resection of bilateral neural foraminal rim enhancing extramedullary lesions and decompression of severe lateral spinal cord compression seen on prior MRI (green arrow); (C) Sagittal postcontrast T1-weighted MRI shows a small amount of abnormal signal in dorsal spinal cord at C7-T1, likely residual edema related to the relieved spinal cord compression (orange arrow); (D) Axial postcontrast T1-weighted MRI shows no remaining bilateral foraminal nerve root compression at C7-T1 (red arrows)

On postoperative day two, post-operative C-spine MRI demonstrated interval C7 and T1 laminectomies with excellent gross total resection of the bilateral neural foraminal rim-enhancing extra-axial lesions and decompression of the severe lateral spinal cord compression (Figure 5). A small amount of abnormal signal was seen in the dorsal spinal cord at the C7-T1 level, most likely edema related to the relieved spinal cord compression (Figure 5). Erythrocyte sedimentation rate was within normal limits (<18mm/h). Antibiotic coverage was narrowed to vancomycin alone, and vancomycin dose was increased to 1.75g every eight hours due to low vancomycin trough concentrations.

On postoperative day four, the patient received a peripherally inserted central catheter (PICC) line to continue IV vancomycin for eight weeks. The patient was discharged on postoperative day six having discontinued all narcotics and antispasmodics and able to ambulate with a rolling walker with assistance.

On postoperative week one, the patient was seen in the outpatient clinic, where she reported significant improvement in leg strength and minimal axial neck tenderness. Strength in bilateral upper extremities was 5/5, while strength in bilateral lower extremities remained 4/5. Physical therapy for continued lower extremity strength improvement was recommended. The patient presented to the clinic with a generalized rash. At the recommendation of the Infectious Disease team, she was switched from IV vancomycin to IV daptomycin 600mg every 24 hours.

After 41 days on daptomycin, the PICC line was discontinued on postoperative day 55 and antibiotics were switched to PO doxycycline for four days to complete a full 8-week course.

At postoperative week 16, the patient had begun walking with a cane instead of a walker, and LE strength was improved to 4+/5 throughout. By postoperative week 19, the patient had regained full bilateral LE strength 5/5. The patient felt her strength was significantly improved and was satisfied with her care.

## Discussion

SSCs are an uncommon cause of radiculopathy and myelopathy [3]. They are defined as fluid-filled collections lined by cyst wall and are found in the vicinity of facet synovial joints [1,4]. Synovial cysts in the spine have been identified more frequently in areas with greater mobility (e.g., L4-L5) and spondylolisthesis and may be products of spinal instability [2,5,6].

SSCs are usually found along with degenerative changes in the lumbosacral facet joints in older individuals, with reported frequencies ranging from 0.6 to 7.3% in patients with myelopathy or radiculopathy [7-10]. The thoracic spine is the second most common location of SSCs [11,12]. In contrast, CSSCs are rare. CSSCs have been classified as cysts occurring in the atlantoaxial junction, and those occurring beneath the atlantoaxial junction (sub-axial CSSCs), including cervicothoracic SSCs. We found 138 reported cases of CSSCs.

We performed a systematic review of previously reported CSSCs (see Appendices) [13]. Based on our review of the literature, sub-axial CSSCs comprise the majority of reported CSSCs (65%); cervicothoracic CSSCs comprised a slight majority of the sub-axial reported CSSCs. Table 1 summarizes all reported cases of CSSCs in the literature.

**Table 1:** Location of Previously Reported Cervical Spinal Synovial Cysts This table summarizes locations and frequencies of CSSC reported during the previous 20 years. Appendix 1 provides information on the methods and articles included in this review.

Location	n (%)
Atlanto-axial	57 (35)
Sub-axial	105 (65)
C2 – C6	47 (29)
C7 – T1 (Cervico-Thoracic)	58 (36)
Total	162 (100)

Pathologic classifications of SSC have been protean. Recently, a classification of SCC based on histological findings was proposed by Chebib et al. at the Massachusetts General Hospital, with SCC being classified as cysts with a synovial lining (true synovial cysts), pseudocysts formed from degenerated ligamentum flavum, and pseudocysts without a synovial lining and without evidence of involvement of the ligamentum flavum [14]. While no synovial cyst lining was observed in our histopathology, confirmatory synovial linings in pathology reports have been sparse in previous case series, with up to 71% of true synovial cysts having incomplete synovial lining [15]. The diagnosis of synovial cysts can be even more challenging when a superimposed infection occurs as was the case in Freedman et al.’s (2010) case report of a 63-year-old man with superinfected synovial cysts of the lumbar spine [16]. To the best of our knowledge, ours and the case reported by Freedman et al. are the only reported cases of superinfected synovial cysts in the literature. Freedman et al. delineate diagnostic clues for identifying infection of synovial cysts, including rapid progression of symptoms and lack of fever. Their patient exhibited a positive culture for Staphylococcus aureus that was managed with culture-specific IV vancomycin for six weeks leading to an almost complete recovery of motor function and resolution of back pain. These findings, including positive S. aureus culture, lack of fever, and rapidly developing motor weakness, closely align with those observed in our case report [16]. Thus, despite a lack of synovial cyst lining in our histopathology specimen, synovial cysts remain our leading differential diagnosis. Bilateral septic arthritis of the facet joints as well as discitis with a cystic inflammatory reaction, or an epidural abscess are considered further down on our differential diagnosis.

Unilateral CSSCs are rare. Two case reports exist; a CSCC at the cervicothoracic junction in which the spinal cord and C8 nerve root were impinged, and a CSCC at C4-C5 in an asymptomatic patient that developed weakness in all extremities and fecal incontinence after a fall [17,18]. SSCs occur very rarely bilaterally. Of 194 patients surgically treated for symptomatic lumbar synovial cysts at Mayo clinic over 24 years, only eight patients presented with bilateral synovial cysts [10]. To the best of our knowledge, only two other case reports of a bilateral CSSCs exist; one in a 64-year-old male at the C7 joint, and one of a 60-year-old male at the atlantoaxial junction [19,20].

## Conclusions

Bilateral CSSCs are a rare cause of myelopathy and radiculopathy, may become superinfected, and require intervention.

## Figures and Tables

**Figure 6:**
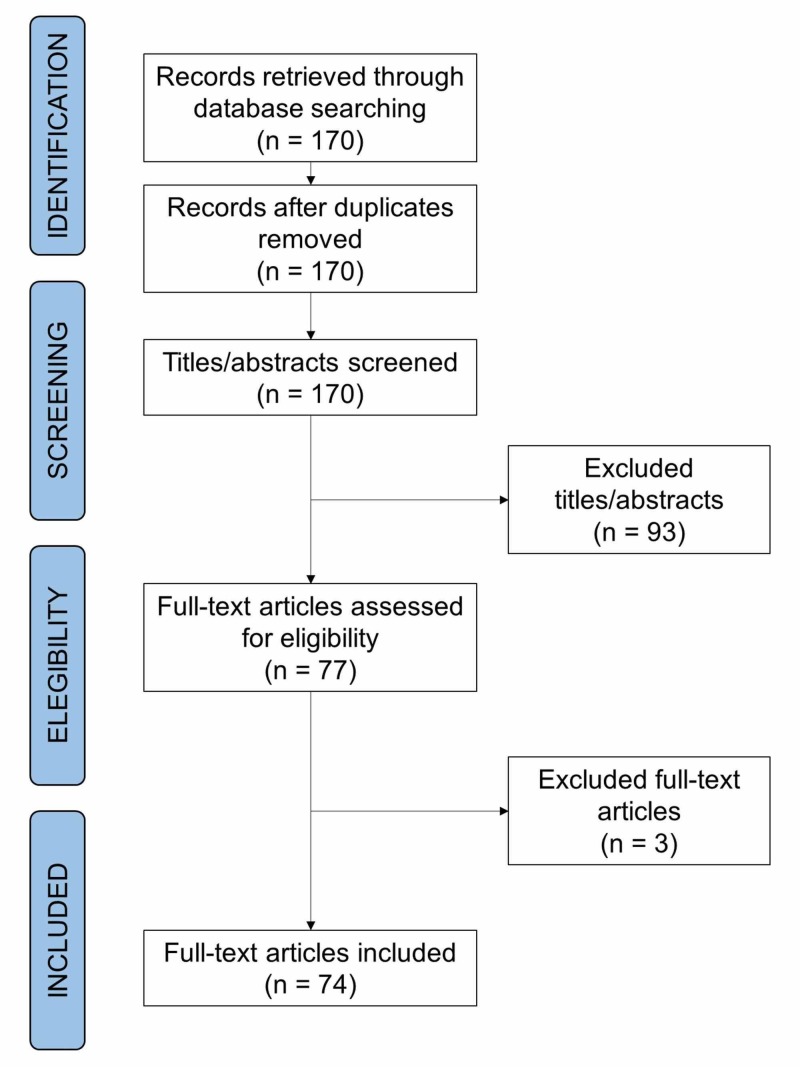
PRISMA Flow Diagram PRISMA flow diagram depicting search strategy used for the systematic review of literature for CSSCs case reports.

## Appendices

Appendix 1. Systematic Review of Previously Reported Cervical Spinal Synovial Cysts

We performed a systematic review of previously reported CSSCs. We used the statement "synovial" OR "ganglion" AND "cyst" AND "cervical" for interrogating PubMed and limited the scope of our review to the latest 20 years (i.e. January 1st, 1999 to December 31st, 2018). We identified 170 records and screened their titles and abstracts for relevance. After eliminating irrelevant titles and abstracts we assessed 77 full-text articles for eligibility and excluded three full-text articles that were not case reports and/or were case reports in non-humans. Ultimately, we included a total of 74 full-text articles in our systematic review (Figure 6). The case reports included in this systematic review are summarized in Table 2.

**Table 2:** Citations of Case Reports Included in Systematic Review

Citation
Cudlip S, Johnston F, Marsh H. Subaxial cervical synovial cyst presenting with myelopathy. Report of three cases. J Neurosurg. 1999 Jan;90(1 Suppl):141-4.
Lunardi P, Acqui M, Ricci G, Agrillo A, Ferrante L. Cervical synovial cysts: case report and review of the literature. Eur Spine J. 1999;8(3):232-7.
Akiyama H, Tamaki N, Kondoh T, Nagashima T. Craniocervical junction synovial cyst associated with atlanto-axial dislocation--case report. Neurol Med Chir (Tokyo). 1999 Jul;39(7):539-43.
Chang H, Park JB, Kim KW. Synovial cyst of the transverse ligament of the atlas in a patient with os odontoideum and atlantoaxial instability. Spine (Phila Pa 1976). 2000 Mar 15;25(6):741-4.
Aksoy FG, Gomori JM. Symptomatic cervical synovial cyst associated with an os odontoideum diagnosed by magnetic resonance imaging: case report and review of the literature. Spine (Phila Pa 1976). 2000 May 15;25(10):1300-2.
Cai CY, Palmer CA, Paramore CG. Exuberant transverse ligament degeneration causing high cervical myelopathy. J Spinal Disord. 2001 Feb;14(1):84-8.
Zorzon M, Skrap M, Diodato S, Nasuelli D, Lucci B. Cysts of the atlantoaxial joint: excellent long-term outcome after posterolateral surgical decompression. Report of two cases. J Neurosurg. 2001 Jul;95(1 Suppl):111-4.
Hatem O, Bedou G, Négre C, Bertrand JL, Camo J. Intraspinal cervical degenerative cyst. Report of three cases. J Neurosurg. 2001 Jul;95(1 Suppl):139-42.
Yamamoto A, Nishiura I, Handa H, Kondo A. Ganglion cyst in the ligamentum flavum of the cervical spine causing myelopathy: report of two cases. Surg Neurol. 2001 Dec;56(6):390-5.
Shima Y, Rothman SL, Yasura K, Takahashi S. Degenerative intraspinal cyst of the cervical spine: case report and literature review. Spine (Phila Pa 1976). 2002 Jan 1;27(1):E18-22.
Giger R, Szalay-Quinodoz I, Haenggeli A, Dulguerov P. Ganglion cyst of the spinal anterior longitudinal ligament presenting as a retropharyngeal mass. Am J Otolaryngol. 2002 Nov-Dec;23(6):390-3.
Ito T, Hayashi M, Ogino T. Retrodental synovial cyst which disappeared after posterior C1-C2 fusion: A case report. J Orthop Surg (Hong Kong). 2000 Jun;8(1):83-87.
Eustacchio S, Trummer M, Unger F, Flaschka G. Intraspinal synovial cyst at the craniocervical junction. Zentralbl Neurochir. 2003;64(2):86-9.
Jost SC, Hsien Tu P, Wright NM. Symptomatic intraosseous synovial cyst in the cervical spine: a case report. Spine (Phila Pa 1976). 2003 Sep 1;28(17):E344-6.
Morio Y, Yoshioka T, Nagashima H, Hagino H, Teshima R. Intraspinal synovial cyst communicating with the C1-C2 facet joints and subarachnoid space associated with rheumatoid atlantoaxial instability. Spine (Phila Pa 1976). 2003 Dec 1;28(23):E492-5.
Cheng YY, Chen CC, Yang MS, Hung HC, Lee SK. Intraspinal extradural ganglion cyst of the cervical spine. J Formos Med Assoc. 2004 Mar;103(3):230-3.
Cho BY, Zhang HY, Kim HS. Synovial cyst in the cervical region causing severe myelopathy. Yonsei Med J. 2004 Jun 30;45(3):539-42.
Miwa M, Doita M, Takayama H, Muratsu H, Harada T, Kurosaka M. An expanding cervical synovial cyst causing acute cervical radiculopathy. J Spinal Disord Tech. 2004 Aug;17(4):331-3.
Fonoff ET, Dias MP, Tarico MA. Myelopathic presentation of cervical juxtafacet cyst: a case report. Spine (Phila Pa 1976). 2004 Dec 1;29(23):E538-41.
Okamoto K, Doita M, Yoshikawa M, Manabe M, Sha N, Yoshiya S. Synovial cyst at the C1-C2 junction in a patient with atlantoaxial subluxation. J Spinal Disord Tech. 2004 Dec;17(6):535-8.
Gazzeri R, Galarza M, Gorgoglione L, Bisceglia M, D'Angelo V. Cervical cyst of the ligamentum flavum and C7-T1 subluxation: case report. Eur Spine J. 2005 Oct;14(8):807-9.
Fiore AJ, Haid RW, Rodts GE, Subach BR, Mummaneni PV, Riedel CJ, Birch BD. Atlantal lateral mass screws for posterior spinal reconstruction: technical note and case series. Neurosurg Focus. 2002 Jan 15;12(1):E5.
McGuigan C, Stevens J, Gabriel CM. A synovial cyst in the cervical spine causing acute spinal cord compression. Neurology. 2005 Oct 25;65(8):1293.
Colen CB, Rengachary S. Spontaneous resolution of a cervical synovial cyst. Case illustration. J Neurosurg Spine. 2006 Feb;4(2):186.
Song JK, Musleh W, Christie SD, Fessler RG. Cervical juxtafacet cysts: case report and literature review. Spine J. 2006 May-Jun;6(3):279-81.
Cheng WY, Shen CC, Wen MC. Ganglion cyst of the cervical spine presenting with Brown-Sequard syndrome. J Clin Neurosci. 2006 Dec;13(10):1041-5.
Christophis P, Asamoto S, Kuchelmeister K, Schachenmayr W. "Juxtafacet cysts", a misleading name for cystic formations of mobile spine (CYFMOS). Eur Spine J. 2007 Sep;16(9):1499-505.
Cecchi PC, Peltz MT, Rizzo P, Musumeci A, Pinna G, Schwarz A. Conservative treatment of an atlantoaxial degenerative articular cyst: case report. Spine J. 2008 Jul-Aug;8(4):687-90.
Kostanian VJ, Mathews MS. CT Guided Aspiration of a Cervical Synovial Cyst. Case Report and Technical note. Interv Neuroradiol. 2007 Sep;13(3):295-8.
Kahiloğullari G, Tuna H, Attar A. Management of spinal synovial cysts. Turk Neurosurg. 2008 Apr;18(2):211-4.
Vastagh I, Palásti A, Nagy H, Veres R, Bálint K, Karlinger K, Várallyay G. Cervical juxtafacet cyst combined with spinal dysraphism. Clin Imaging. 2008 Sep-Oct;32(5):387-9.
Kirk HJ, Pik JH. A novel operative technique to manage a symptomatic synovial cyst associated with an os odontoideum. J Clin Neurosci. 2009 Jun;16(6):822-4.
Marbacher S, Lukes A, Vajtai I, Ozdoba C. Surgical approach for synovial cyst of the atlantoaxial joint: a case report and review of the literature. Spine (Phila Pa 1976). 2009 Jul 1;34(15):E528-33.
Costa F, Menghetti C, Cardia A, Fornari M, Ortolina A. Cervical synovial cyst: case report and review of literature. Eur Spine J. 2010 Jul;19 Suppl 2:S100-2.
Nojiri H, Sakuma Y, Uta S. Degenerative intraspinal cyst of the cervical spine. Orthop Rev (Pavia). 2009 Oct 10;1(2):e17.
Mendes-Araújo L, Rangel C, Domingues RC, Gasparetto EL. Case report. Atlantoaxial synovial cyst causing isolated unilateral hypoglossal nerve paralysis. Br J Radiol. 2010 Feb;83(986):e35-8.
Weng C, Wang LM, Wang WD, Tan HY. Bipartite atlas with os odontoideum and synovial cyst: case report and review literature. Spine (Phila Pa 1976). 2010 May 20;35(12):E568-75.
Muzii VF, Tanganelli P, Signori G, Zalaffi A. Ganglion cyst of the ligamentum flavum: a rare cause of cervical spinal cord compression. A case report. J Neurol Neurosurg Psychiatry. 2010 Aug;81(8):940-1.
Aizawa T, Ozawa H, Kusakabe T, Nakamura T, Chanplakorn P, Itoi E. C1/2 facet cyst revealed by facet joint arthrography. J Orthop Sci. 2010 Jul;15(4):603-7.
Harries A, Wasserberg J. Synovial cyst presenting as a C1/2 tumour. Br J Neurosurg. 2010 Oct;24(5):595-6.
Moon HJ, Kim JH, Kim JH, Kwon TH, Chung HS, Park YK. Cervical juxtafacet cyst with myelopathy due to postoperative instability. Case report. Neurol Med Chir (Tokyo). 2010;50(12):1129-31.
Van Gompel JJ, Morris JM, Kasperbauer JL, Graner DE, Krauss WE. Cystic deterioration of the C1-2 articulation: clinical implications and treatment outcomes. J Neurosurg Spine. 2011 Apr;14(4):437-43.
Lyons MK, Birch BD, Krauss WE, Patel NP, Nottmeier EW, Boucher OK. Subaxial cervical synovial cysts: report of 35 histologically confirmed surgically treated cases and review of the literature. Spine (Phila Pa 1976). 2011 Sep 15;36(20):E1285-9.
Hénaux PL, Hamlat A, Riffaud L, Guégan Y, Morandi X. Spontaneous regression of a symptomatic atlanto-occipital joint cyst. Case report. Neurochirurgie. 2011 Jul;57(3):129-32.
Tofuku K, Koga H, Komiya S. Facet arthrography of a cervical synovial cyst. J Neurointerv Surg. 2012 Jul;4(4):e17.
Found E, Bewyer D. Cervical synovial cyst: case report. Iowa Orthop J. 2011;31:215-8.
Takeuchi M, Yasuda M, Takahashi E, Funai M, Joko M, Takayasu M. A large retro-odontoid cystic mass caused by transverse ligament degeneration with atlantoaxial subluxation leading to granuloma formation and chronic recurrent microbleeding case report. Spine J. 2011 Dec;11(12):1152-6.
Lyons MK, Birch B. Transoral surgical approach for treatment of symptomatic atlantoaxial cervical synovial cysts. Turk Neurosurg. 2011;21(4):483-8.
Parks RM, König MA, Boszczyk B, Shafafy M. Transarticular fusion for treatment of cystic lesion arising from an odontoid fracture. Eur Spine J. 2013 Jan;22(1):21-5.
Schmitz MR, Jenné J. Acute Tetraparesis Caused by a Cervical Spine Synovial Cyst Associated with an Os Odontoideum: A Case Report. JBJS Case Connect. 2012 Apr-Jun;2(2):e17.
Machino M, Yukawa Y, Ito K, Kato F. Cervical degenerative intraspinal cyst: a case report and literature review involving 132 cases. BMJ Case Rep. 2012 Nov 28;2012.
Sameshima T, Shibahashi K, Nozaki T, Akabane A, Kihara A, Horiuchi H, Morita A. Atlantoaxial intraspinal juxtafacet cyst. Neurol Med Chir (Tokyo). 2013;53(2):125-8.
Pikis S, Cohen JE, Barzilay Y, Hasharoni A, Kaplan L, Itshayek E. Symptomatic facet cysts of the subaxial cervical spine. J Clin Neurosci. 2013 Jul;20(7):928-32.
Sasamori T, Hida K, Anzai K, Yano S, Kato Y, Tanaka S, Saito H, Houkin K. A case of cervical juxtafacet cyst with extensive rim enhancement on Gd-DTPA MRI. Clin Imaging. 2014 Mar-Apr;38(2):199-201.
Conforti G, Della Pepa GM, Papacci F, Scerrati A, Montano N. Hemorrhagic synovial cyst as an 'evanescing' spinal cervical mass: an issue for differential diagnosis. Acta Neurol Belg. 2014 Dec;114(4):325-7.
Sheen JJ, Seo DK, Rhim SC, Choi SH. Hemorrhagic synovial cyst associated with rheumatoid atlantoaxial subluxation. Korean J Spine. 2013 Jun;10(2):85-7.
Attwell L, Elwell VA, Meir A. Cervical synovial cyst. Br J Neurosurg. 2014 Dec;28(6):813-4.
Kim SW, Ju CI, Kim HS, Kim YS. Brown-séquard syndrome caused by a cervical synovial cyst. J Korean Neurosurg Soc. 2014 Apr;55(4):215-7.
Bandín-Diéguez FJ, Pita-Buezas L, Vázquez-Herrero F, Gelabert-González M. [Spinal cord compression secondary to a cervical synovial cyst]. Rev Neurol. 2014 Oct 1;59(7):327-8.
Bydon M, Lin JA, de la Garza-Ramos R, Sciubba DM, Wolinsky JP, Witham TF, Gokaslan ZL, Bydon A. The role of spinal fusion in the treatment of cervical synovial cysts: a series of 17 cases and meta-analysis. J Neurosurg Spine. 2014 Dec;21(6):919-28.
Kim DS, Yang JS, Cho YJ, Kang SH. Acute myelopathy caused by a cervical synovial cyst. J Korean Neurosurg Soc. 2014 Jul;56(1):55-7.
Colasanti R, Lamki T, Tailor AR, Ammirati M. Recurrent atlantoaxial synovial cyst resection via a navigation-guided, endoscope-assisted posterior approach. Surg Neurol Int. 2014 Dec 30;5(Suppl 15):S567-9.
Ikegami D, Matsuoka T, Aoki Y. Immediate Reduction of a Retro-odontoid Synovial Cyst Following Lateral Atlantoaxial Joint Puncture and Arthrography: A Case Report. Spine (Phila Pa 1976). 2015 May 15;40(10):E609-12.
Overvliet G, van Scherpenzeel-de Vries MA, Wattjes MP, Vermeulen RJ. Cervical Synovial Cyst in a 16-Year-Old Girl. Pediatr Neurol. 2015 Aug;53(2):173-4.
Corredor JA, Quan G. Cervical Synovial Cyst Causing Cervical Radiculomyelopathy: Case Report and Review of the Literature. Global Spine J. 2015 Aug;5(4):e34-8.
Breckwoldt T, Oktenoglu T, Sasani M, Suzer T, Ozer AF. A rare cause of root-compression: Subaxial cervical synovial cyst in association with congenital fusion. Int J Surg Case Rep. 2015;16:90-2.
Phan K, Mobbs RJ. A rare case of cervical facet joint and synovial cyst at C5/C6. J Clin Neurosci. 2016 Jul;29:191-4.
Theodotou CB, Urakov TM, Vanni S. Atlantoaxial Synovial Cyst: Case Report and Literature Review. World Neurosurg. 2016 Aug;92:588.e7-588.e15.
Hartmann S, Tschugg A, Kavakebi P, Thomé C. Intradural synovial cyst of the atlantoaxial joint: a case report. Acta Neurochir (Wien). 2016 Aug;158(8):1583-6.
Tangviriyapaiboon T. Complete Spontaneous Regression of the Intraspinal Synovial Cyst at the C1-C2 Junction Following with Atlantoaxial Fusion of Non-Union Odontoid Fracture: A Case Report. J Med Assoc Thai. 2016 Jun;99 Suppl 3:S120-5.
Linhares D, Lobo J, Pinto R, Neves N. Atypical presentation of a cervical synovial cyst. Eur Spine J. 2017 Sep;26(9):2267-2271.
Kim J, Choi JG, Son BC. Bilateral Ganglion Cysts of the Ligamentum Flavum in the Cervical Spine Causing a Progressive Cervical Radiculomyelopathy and Literature Review. Case Rep Neurol Med. 2017;2017:3953641.
Ruetten S, Hahn P, Oezdemir S, Baraliakos X, Godolias G, Komp M. Surgical treatment of cervical subaxial intraspinal extradural cysts using a full-endoscopic uniportal posterior approach. J Orthop Surg (Hong Kong). 2018 May-Aug;26(2):2309499018777665.
Themistoklis KM, Papasilekas TI, Boviatsis KA, Giakoumettis DA, Vlachakis EN, Themistocleous MS, Sakas DE, Korfias SI. Spinal synovial cysts. A case series and current treatment options. J Clin Neurosci. 2018 Nov;57:173-177.
